# Results and adverse events of personalized peptide receptor radionuclide therapy with ^90^Yttrium and ^177^Lutetium in 1048 patients with neuroendocrine neoplasms

**DOI:** 10.18632/oncotarget.24524

**Published:** 2018-02-15

**Authors:** Richard P. Baum, Harshad R. Kulkarni, Aviral Singh, Daniel Kaemmerer, Dirk Mueller, Vikas Prasad, Merten Hommann, Franz C. Robiller, Karin Niepsch, Holger Franz, Arthur Jochems, Philippe Lambin, Dieter Hörsch

**Affiliations:** ^1^ THERANOSTICS Center for Molecular Radiotherapy, Zentralklinik Bad Berka GmbH, Bad Berka, Germany; ^2^ Department of General and Visceral Surgery, Zentralklinik Bad Berka GmbH, Bad Berka, Germany; ^3^ Clinic for Nuclear Medicine, Charité, Berlin, Germany; ^4^ Center of Molecular Imaging, Zentralklinik Bad Berka GmbH, Bad Berka, Germany; ^5^ Lohmann and Birkner, Berlin, Germany; ^6^ Department of Radiology, GROW - School for Oncology and Developmental Biology, Maastricht University Hospital, Maastricht, The Netherlands; ^7^ Department of Radiation Oncology (The D-Lab), GROW - School for Oncology and Developmental Biology, Maastricht University, Maastricht, The Netherlands; ^8^ Department of Gastroenterology/Endocrinology, Center for Neuroendocrine Tumors Bad Berka – ENETS Center of Excellence, Zentralklinik Bad Berka GmbH, Bad Berka, Germany

**Keywords:** peptide receptor radionuclide therapy, neuroendocrine tumors, survival, functional syndromes

## Abstract

**Introduction:**

Peptide receptor radionuclide therapy (PRRT) of patients with somatostatin receptor expressing neuroendocrine neoplasms has shown promising results in clinical trials and a recently published phase III study.

**Methods:**

In our center, 2294 patients were screened between 2004 and 2014 by ^68^Ga somatostatin receptor (SSTR) PET/CT. Intention to treat analysis included 1048 patients, who received at least one cycle of ^90^Yttrium or ^177^Lutetium-based PRRT. Progression free survival was determined by ^68^Ga SSTR-PET/CT and EORTC response criteria. Adverse events were determined by CTCAE criteria.

**Results:**

Overall survival (95% confidence interval) of all patients was 51 months (47.0-54.9) and differed significantly according to radionuclide, grading, previous therapies, primary site and functionality. Progression free survival (based on PET/CT) of all patients was 19 months (16.9-21), which was significantly influenced by radionuclide, grading, and origin of neuroendocrine neoplasm. Progression free survival after initial progression and first and second resumption of PRRT after therapy-free intervals of more than 6 months were 11 months (9.4-12.5) and 8 months (6.4-9.5), respectively. Myelodysplastic syndrome or leukemia developed in 22 patients (2.1%) and 5 patients required hemodialysis after treatment, other adverse events were rare.

**Conclusion:**

PRRT is effective and overall survival is favorable in patients with neuroendocrine neoplasms depending on the radionuclide used for therapy, grading and origin of the neuroendocrine neoplasm which is not exactly mirrored in progression free survival as determined by highly sensitive ^68^Ga somatostatin receptor PET/CT using EORTC criteria for determining response to therapy.

## INTRODUCTION

Neuroendocrine neoplasms (NENs) arise most frequently in the gastroentero-pancreatic system and lungs. These neoplasms originate in scattered endocrine cells of the diffuse neuroendocrine system and are biologically very heterogeneous [1]. Two groups of NEN behave biologically and clinically distinctively different; the well differentiated slowly growing neuroendocrine tumors with an excellent to good prognosis and poorly differentiated fast growing neuroendocrine carcinomas with a poor prognosis. Grading of NENs is performed by Ki67 index. G1 and G2 neuroendocrine tumors have a Ki67 index of 20% or below whereas neuroendocrine carcinomas have a Ki 67 index of more than 20%. Neuroendocrine carcinomas are defined by small or large cell morphology. In addition, the new WHO classification of pancreatic NENs includes the newly created well differentiated neuroendocrine tumors with a proliferation of up to 50% as neuroendocrine tumor G3 [2, 3]. NENs may be functionally active due to the autonomous secretion of biogenic amines or peptide hormones such as serotonin, gastrin or insulin causing characteristic functional syndromes [4].

The majority of well-differentiated NENs overexpress receptors of the 5 subclasses of somatostatin receptors (SSTRs), most frequently receptors 2 and 5. Activated SSTRs have anti-secretory and anti-proliferative activity, and are targeted by stable somatostatin analogues for treatment of functional syndromes and to reduce tumor growth of NENs [5–7]. In addition, SSTR are utilized to visualize NENs by molecular imaging. SSTR scintigraphy uses stable somatostatin analogues coupled to short-lived radionuclides such as ^111^Indium or ^99m^Technetium. The more recently developed SSTR PET/CT involves ^68^Gallium (^68^Ga) as a positron emitter, coupled to stable somatostatin analogues. This technique is more sensitive, faster with much lesser radiation exposure, compared to the standard SSTR scintigraphy [8]. Netspot, the first kit for the preparation of ^68^Ga-DOTATATE for PET/CT imaging of NENs was recently approved by the US FDA (http://www.fda.gov/Drugs/InformationOnDrugs/ucm508921.htm).

Coupling beta-and/or gamma-emitting radionuclides to stable somatostatin analogues permits internal radiation of SSTR-expressing NENs by peptide receptor radionuclide therapy (PRRT). ^90^Yttrium (^90^Y) and ^177^Lutetium (^177^Lu) are used as radionuclides and chelated to stable somatostatin analogues such as DOTATATE and DOTATOC [9, 10]. Numerous phase I and phase II studies have shown favorable progression-free survival (PFS) and overall survival (OS) in NEN patients compared to historical controls [9–11]. Recently, a phase III study (NETTER-1 trial) has compared 4 cycles of PRRT, each using 7.4 GBq of ^177^Lu-DOTATATE (Lutathera®) together with 30 mg Octreotide-LAR every 4 weeks, to high dose somatostatin analogue therapy with 2 × 30 mg Octreotide-LAR every 4 weeks. Patients with progressive NENs of small bowel under treatment with somatostatin analogues and with SSTR expression were included, and 230 were randomized in a 1:1 ratio. In the control group, median PFS was 8.4 months, whereas it was not reached yet in the Lutathera® group (estimation 40 months). The hazard ratio was 0.209 (95% CI, 0.129-0.33) in favor of Lutathera®. Adverse events were rare and the treatment was well tolerated [12].

**Figure 1:**
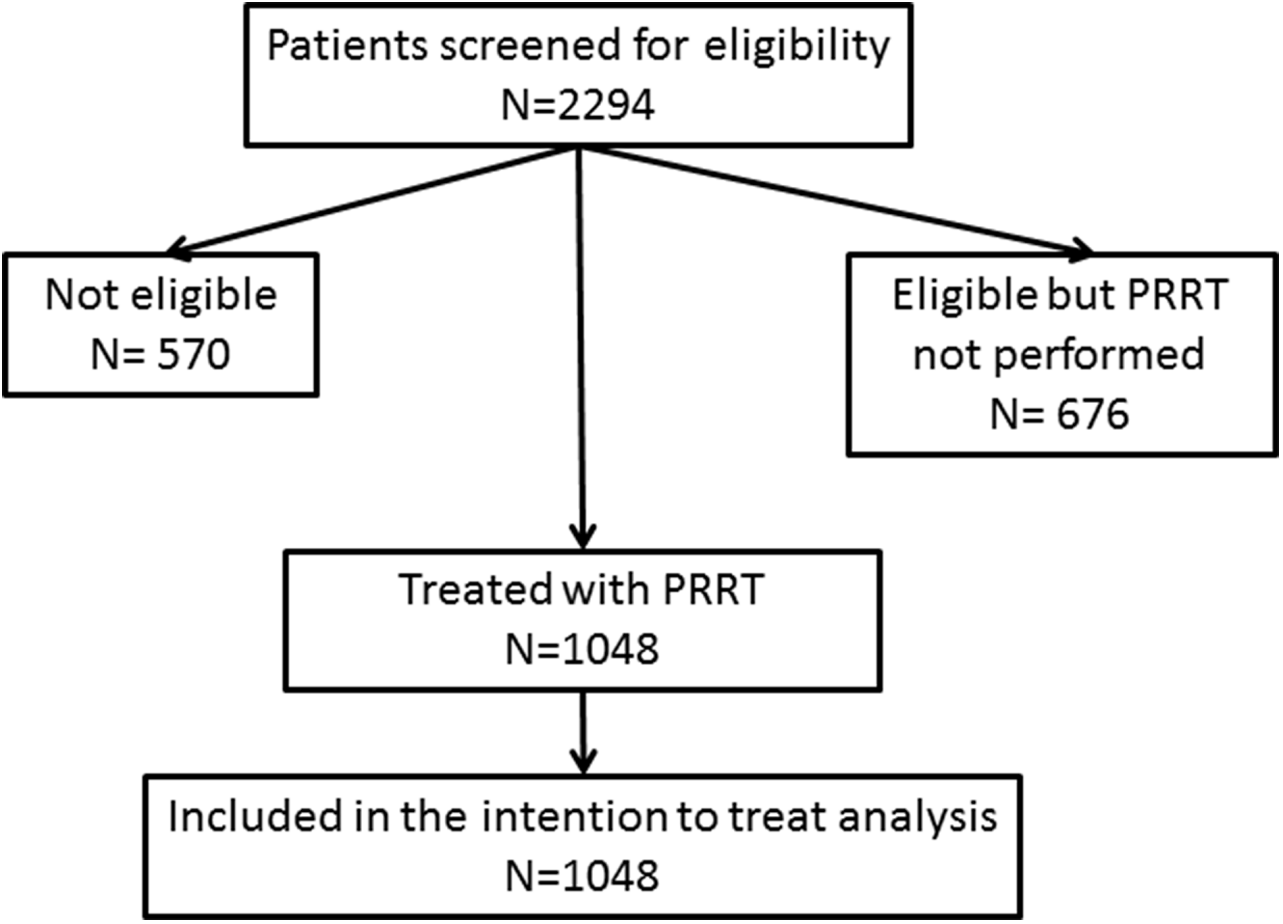
Flow chart of trial characteristics

Standardized uptake values (SUVs) of SSTR PET/CT predict absorbed dose and response to PRRT [13–16]. Here, we present OS and PFS in 1048 patients treated with at least one cycle of PRRT using ^90^Y or ^177^Lu, which were selected and re-staged by ^68^Ga-SSTR PET/CT (Figure 1). Response to PRRT evaluated by radiological criteria (RECIST 1.1) has been reported previously in two subgroups of 56 and 200 patients, respectively [11, 17].

## RESULTS

### Patient characteristics

The majority of patients (593/1048) were males (56.6%) and 455 (43.4%) were females. Most patients (559/1048) were between 41 and 60 years at diagnosis (53.3%) whereas 393 (37.5%) were older than 60 years. At diagnosis, 96 patients (9.2%) were 40 years or younger. This group included 1 patient less than 10 years of age, 5 patients between 11 and 20 years, 24 patients between 21 and 30 and 76 patients aged between 31 and 40 years. Most patients received PRRT with ^177^Lu; 378 as monotherapy (36.1%) and 513 (48.9%) in combination with ^90^Yttrium either as a combination of both radionuclides in one cycle (TANDEM) or sequential (DUO). Only 157 patients (15%) received ^90^Y-DOTATATE or -DOTATOC as monotherapy. Mean administered radioactivity was 18.84 GBq with a minimum of 1.4 GBq and a maximum of 63.9 GBq. Most patients (247 and 399, respectively) had well differentiated NENs of grade 1 (23.6%) or grade 2 (38.1%). A minority of 67 patients (6.4%) had well differentiated NENs grade 3. Grading was not available in 335 patients (31.9%), which were diagnosed before WHO criteria were published and no tissue was available anymore for analysis. These patients were included as a separate group. The majority of patients had been pretreated by one or more other therapies (surgery, somatostatin analogs, chemotherapy, molecular therapy). PRRT was performed as first-line treatment in 119 patients (11.4%) with mostly ileum NENs. More than 3 other therapies before PRRT had been performed in 209 patients (19.9%), whereas 417 patients (39.8%) had received 2-3 therapies and 303 (28.9%) had had one therapy. Primary tumors were localized in pancreas (384 or 36.6%), small intestine (315 or 30.1%), lung (75 or 7.2%), colon and rectum (52 or 5.0%), duodenum (22 or 2.1%), thymus and mediastinum (16 or 1.5%) stomach (15 or 1.4%), caecum and appendix (5 or 0.5%), others (13 or 1.2%) and 151 patients (14.4%) had tumors with unknown primary site. Other primary tumors included paragangliomas/pheochromocytomas (15 patients) and 25 patients with medullary thyroid cancer. For statistical analysis, the group of others included other primary tumor as described above and primaries of duodenum, stomach, thymus, mediastinum, stomach, caecum and appendix.

Functional syndromes were present in 244 patients (23.3%), whereas 804 (76.7%) had no symptoms of hormone overproduction. Carcinoid syndrome was predominant (158 or 15.0%), followed by Zollinger-Ellison syndrome/gastrinoma (46 or 4.4%), the syndromes caused by glucagonoma (16 or 1.5%), insulinoma (15 or 1.4%) and the Werner-Morrison syndrome caused by VIPoma (8 or 0.7%).

### Overall survival

**Figure 2:**
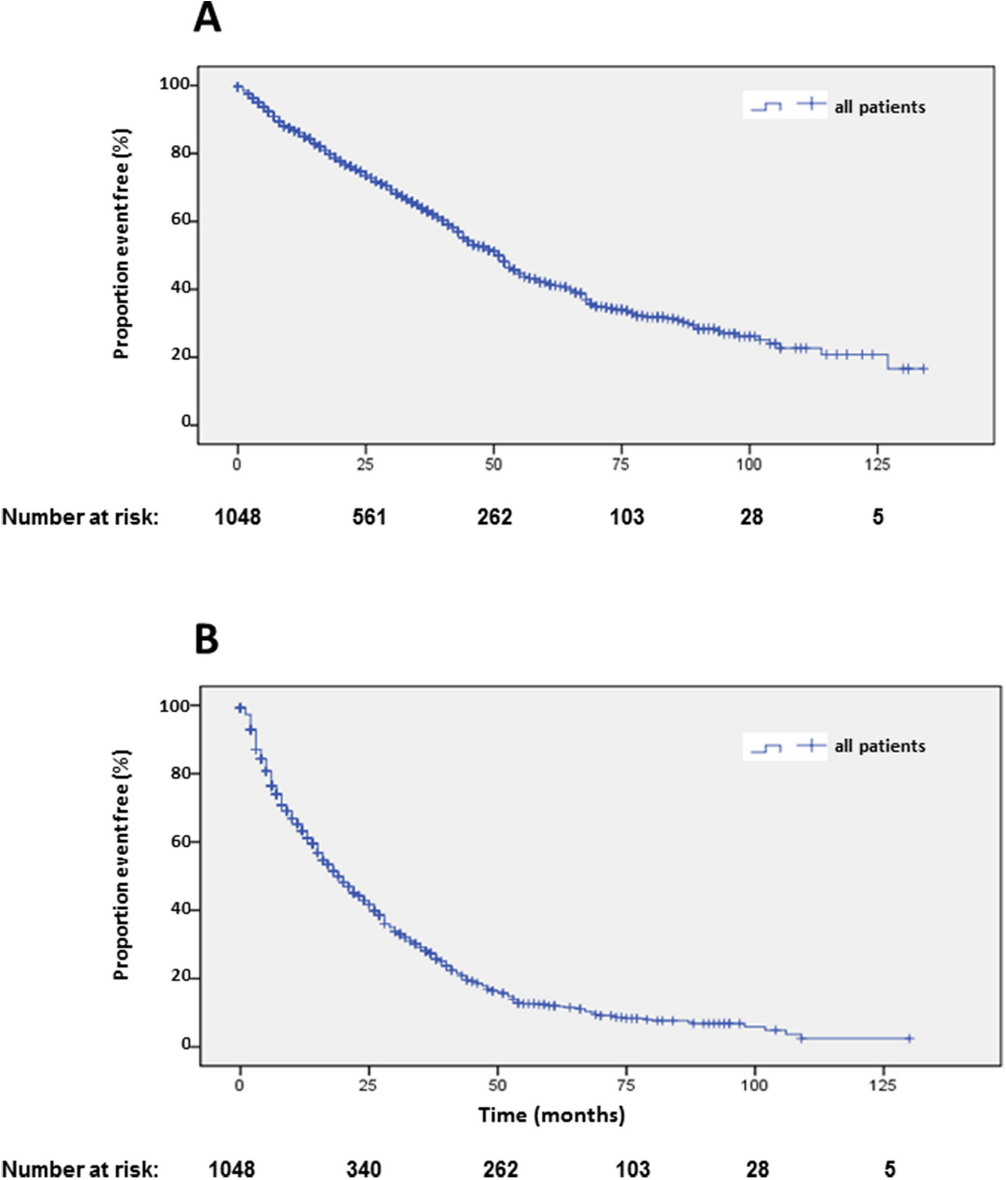
Kaplan-Meier plots of overall survival **(A)** and progression-free survival 1 **(B)** after start of PRRT.

**Figure 3:**
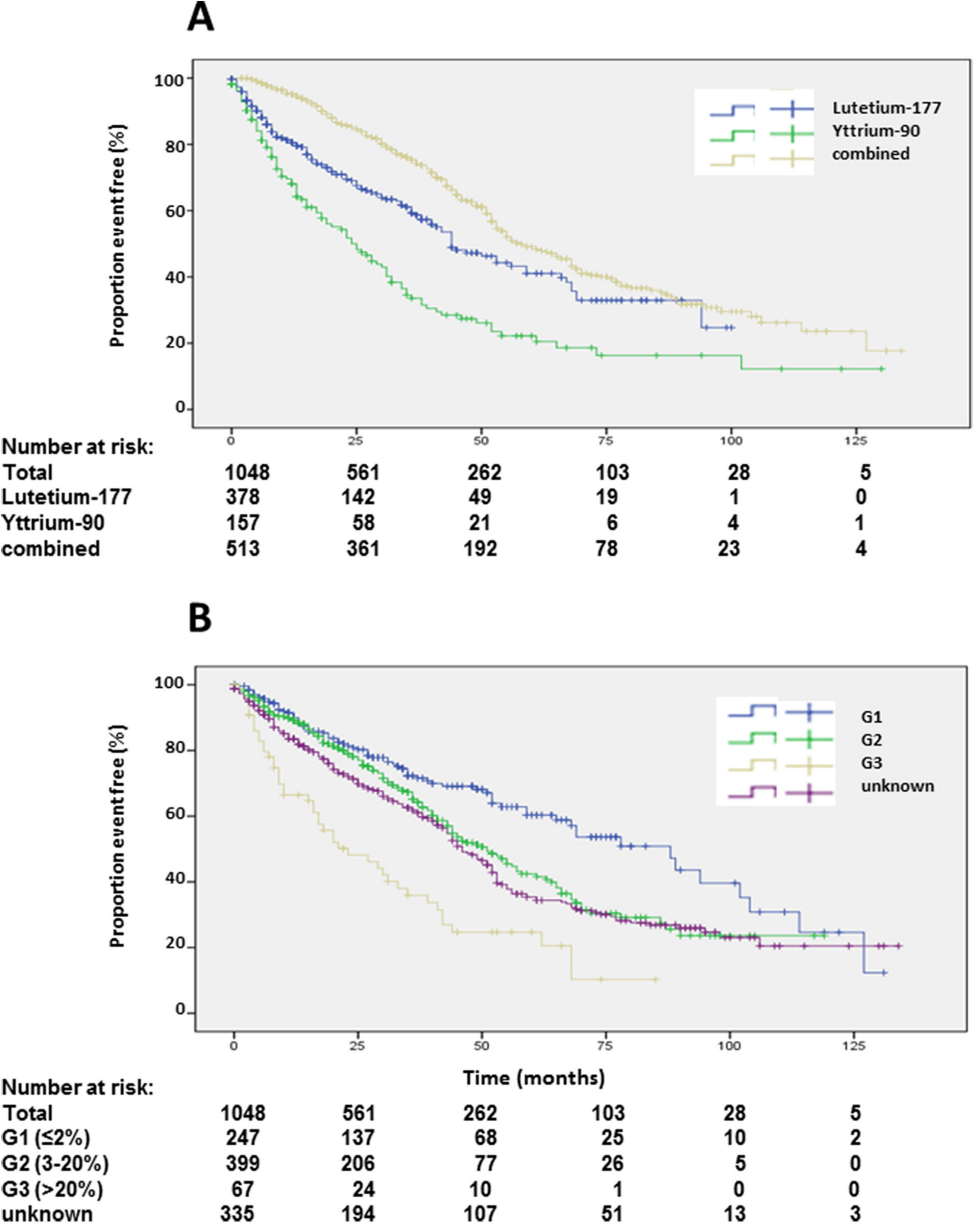
Kaplan-Meier plots of overall survival according to radioisotope **(A)** and grading **(B)**.

Data and statistical analysis on overall survival (OS) is presented in Table 1. Of 1048 patients, 573 (54.7%) were alive with a median OS of 51 months (Figure 2A). Univariate analysis suggested a significant difference in OS for female gender, which was not corroborated by multivariate analysis (Table 1, Supplementary Figure 1A). Compared to the main age group at diagnosis between 40 and 60 years, patients older than 60 years at diagnosis had a significantly shorter survival. Longer survival of patients younger than 40 years at diagnosis was not statistically significant in multivariate analysis (Table 1, Supplementary Figure 1B). Best OS was achieved by a combination of ^90^Y- and ^177^Lu- for PRRT. Shortest survival was observed in patients treated exclusively with ^90^Y, while treatment with solely ^177^Lu resulted in an intermediate survival between the two extreme curves. These differences were highly statistically significant in univariate and multivariate analysis (Table 1, Figure 3A). Grading of NENs was a strong predictor of overall survival. Best overall survival was achieved in the G1 group, followed by G2. G3 NENs had the shortest overall survival with 23 months compared to 88 months in the G1 group. Compared to G2, overall survival of G1 patients was significantly longer and significantly shorter in the G3 group. The group of patients with G3 NENs included 18 patients with a proliferation rate of more than 50%. These patients had a median overall survival of 8.7 months (95% confidence interval 0-21.1).

**Table 1:** Patient characteristics and results of overall survival after start of PRRT

Analysis	Number	%	Death patients	Median	95% CI	Univariate analysis		Multivariate analysis	
						p	HR	95% CI	p
**All Patients**	1048	100	475	51	47.0-54.9				
**Gender**									
Male	593	56.6	276	50	45.4-54.5	**0.029**	1		
Female	455	43.4	199	53	47.3-58.6		0.84	0.7-1.02	0.07
**Age**									
≤40 years	96	9.1	40	70	33.3-106.6	**0.018**	0.91	0.65-1.28	0.6
>40 and ≤60 years	559	53.4	248	54	46.6-61.3		1		
>60 years	393	37.5	187	46	41.8-50.2		1.32	1.08-1.61	**0.01**
**Radionuclide**									
Lutetium-177	378	36	143	44	36-1-52	**<0.001**	1.67	1.33-2.08	**<0.001**
Yttrium-90	157	15	100	24	17.4-30.6		2.89	2.27-3.69	**<0.001**
combined	513	49	232	64	51.7-64.3		1		
**Grading**									
G1 (Ki67<2%)	247	23.5	76	88	69.3-106.6	**<0.001**	0.65	0.49-0.86	**0.0025**
G2 (Ki67 3-20%)	399	38.1	162	51	44-57.9		1		
G3 (Ki67>20%)	67	6.4	22	23	10.8-35.2		1.71	1.21-2.4	**0.0023**
unknown	335	32	143	46	41.5-50.4		1.04	0.84-1.29	0.74
**Previous therapies**									
0	119	11.4	23	55	37.4-72.5	**<0.001**	0.72	0.46-1.12	0.15
1	303	29	119	62	52.1-71.8		0.7	0.55-0.88	**0.0028**
2-3	417	39.7	218	51	45-56.9		1		
>3	209	19.9	115	41	35.3-46.6		1.39	1.1-1.76	**0.01**
**Primary tumor**									
Bronchial	75	7.2	41	40	30.5-49.4	**0.01**	1.08	0.76-1.54	0.67
Pancreas	384	36.7	200	44	37.7-50.2		1		
Small Intestine	315	30	120	69	53.7-84.2		0.57	0.44-0.75	**<0.001**
CUP	151	14.4	60	53	37.5-68.4		0.78	0.58-1.06	0.11
Other	123	11.7	54	47	38.8-55.1		0.7	0.51-0.95	**0.02**
**Functional syndromes**									
Carcinoid syndrome	158	15	82	49	38.7-59.2	**0.012**	1.17	0.89-1.52	0.26
Gastrinoma	46	4.3	21	66	48.9-83		0.8	0.51-1.26	0.34
Insulinoma	15	1.4	10	32	9.7-54.2		1.06	0.56-2.03	0.86
Glucagonoma	16	1.5	7	127	nr		0.65	0.3-1.4	0.27
VIPoma	8	0.7	3	46	14.6-77.3		0.78	0.25-2.46	0.68
Other	1	0.1	1	52	nr		5.64	0.87-36.6	0.07
None	804	76.7	351	51	47-54.9		1		

Abbreviations: nr: not reached; 95 CI: 95% confidence interval bold figures indicate significant results.

**Figure 4:**
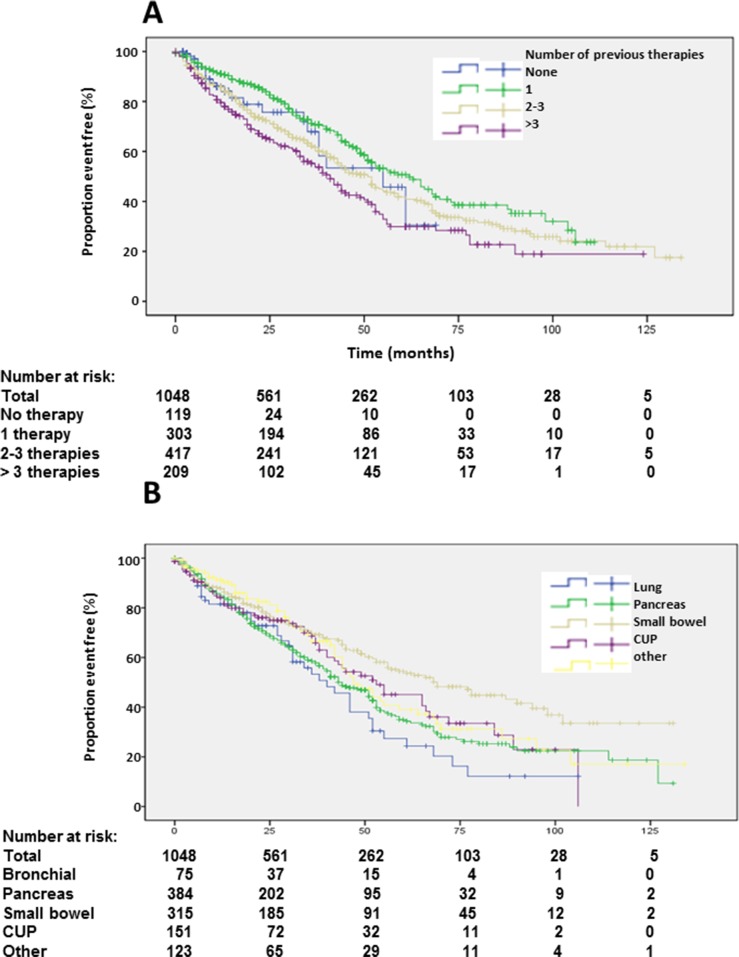
Kaplan-Meier plots of overall survival according to number of previous therapies **(A)** and primary tumors **(B)**.

Patients with unknown grading had similar overall survival as G2 patients (Table 1, Figure 3B). Patients with more than 3 prior therapies had significantly lower survival whereas patients with only one previous therapy had a significantly longer survival (Table 1, Figure 4A). Site of origin of NENs was a predictor of median overall survival in one group only. Best survival was observed in patients with NENs of small bowel (69 months), which was statistically significant followed by CUP, others, pancreas and lung. In between the later groups, differences did not achieve statistical significance except for the heterogeneous group of other neuroendocrine neoplasias (Table 1, Figure 4B). This group included 25 patients with medullary thyroid cancer. These patients had a median overall survival of 32 months (23.8-43.5).

The majority of patients had no functional syndrome and a median overall survival of 51 months. Patients with carcinoid syndrome had a slightly lower median overall survival of 49 months whereas patients with gastrinomas and glucagonomas had longer median overall survival of 66 and 127 months, respectively. Patients with insulinomas had a shorter median overall survival of 32 months whereas patients with VIPomas were similar to non-functional patients or patients with carcinoid syndrome with a median overall survival of 46 months (Table 1, Supplementary Figure 3A).

### Progression-free survival

Patients were restaged by ^68^Ga-SSTR PET/CT and tumor status was determined by RECIST and EORTC criteria. In addition, progression was defined as resumption of PRRT after an interval of more than 6 months or of any other therapy such as surgery, chemotherapy, local ablative therapies or molecular therapies according to the decision of the tumor board. As such, we determined progression-free survival after at least one cycle of PRRT (PFS1) including all 1048 patients (Table 2). Resumption of PRRT (2nd phase) was performed in 470 patients (PFS2; Supplementary Table 1) and again (3rd phase) in 184 patients (PFS3; Supplementary Table 2).

**Table 2:** Patient characteristics and results of progression-free survival after start of PRRT

	Number	%	Progressive patients	Median	95% CI	Univariate analysis		Multivariate analysis	
						p	HR	95% CI	p
**All Patients**	1048	100	774	19	16.9-21				
**Gender**									
Male	593	56.6	447	18	15.9-20	0.108	1		
Female	455	43.4	327	22	18.8-25.1		0.87	0.75-1	0.05
**Age**									
≤40 years	96	9.1	68	18	13.9-22	0.281	0.92	0.71-1.19	0.51
>40 and ≤60 years	559	53.4	420	21	17.7-24.2		1		
>60 years	393	37.5	286	19	16.4-21.5		1.04	0.89-1.22	0.64
**Radionuclide**									
Lutetium-177	378	36	236	17	14-19.9	**<0.001**	1.12	0.94-1.33	0.20
Yttrium-90	157	15	122	13	9.8-16.1		1.41	1.15-1.74	**0.001**
combined	513	49	416	24	21.2-26.7		1		
**Grading**									
G1 (Ki67<2%)	247	23.5	162	22	16.6-27.3	**<0.001**	0.86	0.71-1.05	0.14
G2 (Ki67 3-20%)	399	38.1	292	21	18.1-23.9		1		
G3 (Ki67>20%)	67	6.4	58	7	5-8.9		1.72	1.29-2.3	**<0.001**
unknown	335	32	262	19	15.1-22.8		0.91	0.77-1.08	0.3
**Previous therapies**									
0	119	11.4	45	24	8.3-39.6	**<0.001**	0.78	0.57-1.08	0.13
1	303	29	231	22	18.8-25.1		0.91	0.77-1.09	0.32
2-3	417	39.7	334	18	14.8-21.1		1		
>3	209	19.9	164	17	12.4-21.5		1.18	0.97-1.43	0.10
**Primary tumor**									
Bronchial	75	7.2	62	11	6.3-15.6	**<0.001**	1.43	1.08-1.91	**0.01**
Pancreas	384	36.7	303	20	17-22.9		1		
Small Intestine	315	30	211	22	17.6-26.3		0.75	0.61-0.92	**0.01**
CUP	151	14.4	115	13	9.5-16.4		1.27	1.01-1.58	**0.04**
Other	123	11.7	83	20	13.9-26		0.83	0.64-1.06	0.14
**Functional syndromes**									
Carcinoid syndrome	158	15	128	16	12.6-19.3	0.612	1.18	0.95-1.46	0.13
Gastrinoma	46	4.3	39	26	19.1-32.8		1.01	0.72-1.42	0.95
Insulinoma	15	1.4	13	28	12.5-43.4		0.95	0.54-1.66	0.86
31Glucagonoma	16	1.5	13	20	nr-40.1		0.94	0.54-1.66	0.85
VIPoma	8	0.7	6	22	14.6-29.3		0.79	0.35-1.79	0.59
Other	1	0.1	1	16	nr		1.96	0.27-14.3	0.52
None	804	76.7	574	19	16.9-21		1		

Abbreviations: nr: not reached; 95 CI: 95% confidence interval bold figures indicate significance results.

**Figure 5:**
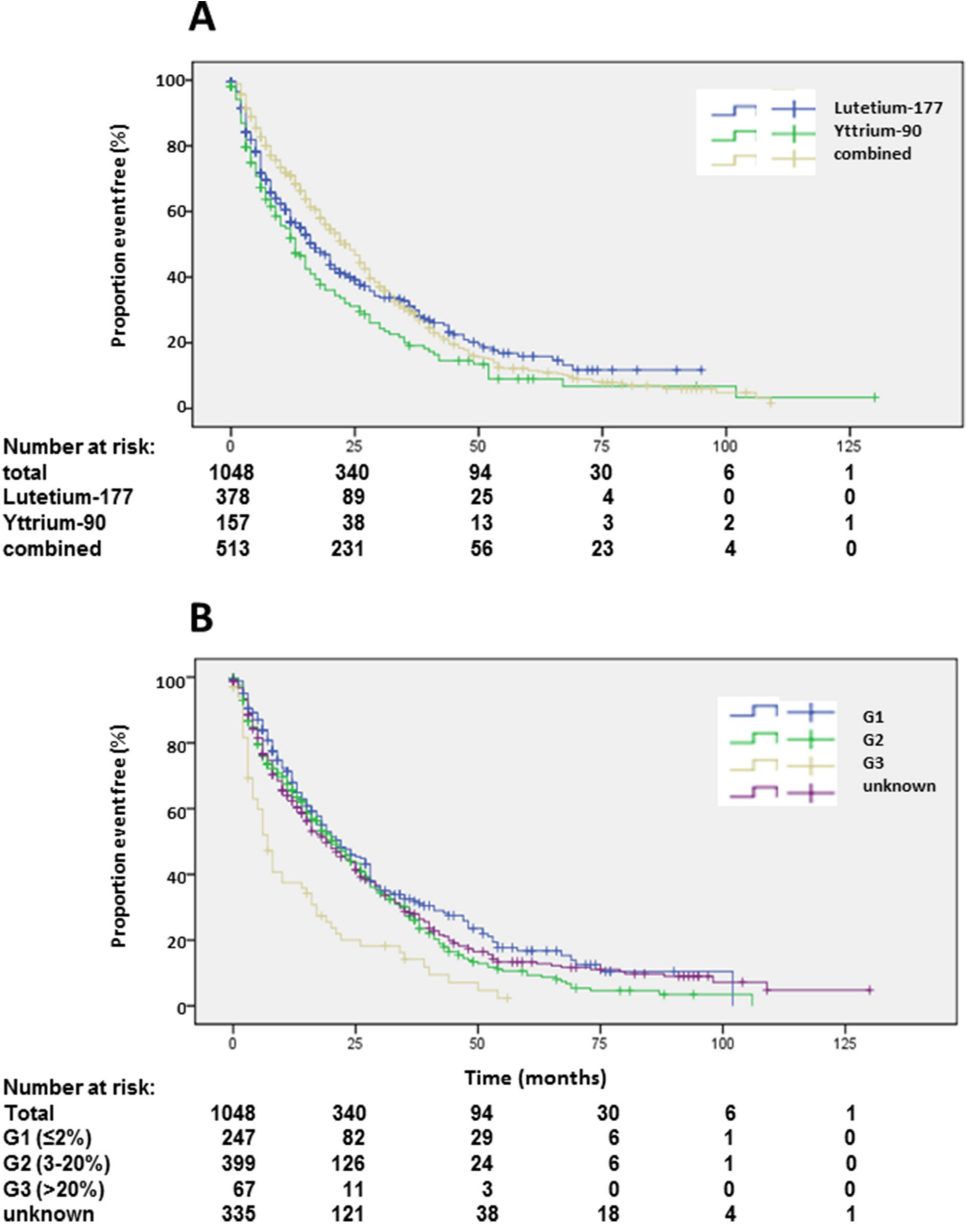
Kaplan-Meier plots of progression-free survival 1 according to radioisotope **(A)** and grading **(B)**.

**Figure 6:**
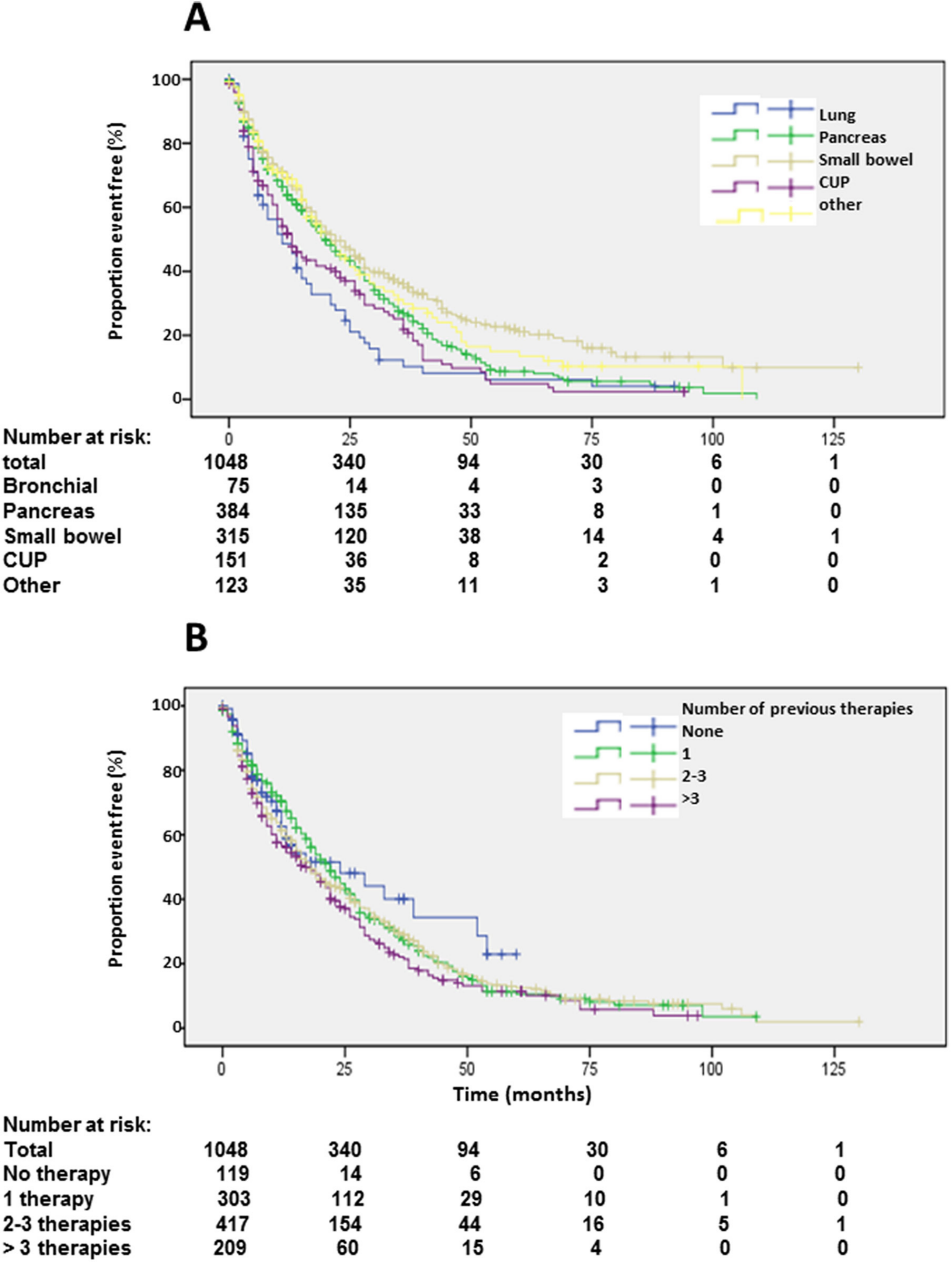
Kaplan-Meier plots of progression-free survival 1 according to primary tumors **(A)** and number of previous therapies **(B)**.

Median PFS1 based on the EORTC PET criteria was 19 months (Figure 2B). Detailed subgroup and statistical analysis of PFS1 is depicted in Table 2. Uni-variate analysis depicted statistically significant differences according to radionuclides (Figure 5A), grading (Figure 5B), number of previous therapies (Figure 6B) and origin of primary tumors (Figure 6A) but not gender (Supplementary Figure 2A), age (Supplementary Figure 2B) and functionality (Supplementary Figure 3B) (Table 2). Multi-variate analysis revealed that patients treated with ^90^Y exclusively had a significantly shorter PFS than patients treated with ^177^Lu or a combination of both radionuclides (Table 2, Figure 5A). Grading determined a significantly lower PFS only in patients with G3 tumors, but there was no significant difference in PFS between G1 and G2 tumors, or between G2 tumors and tumors with unknown proliferation rate (Figure 5B, Table 2). Patients with G3 NENs with more than 50% proliferation rate had a median PFS1 of 5.7 months (nr-12.1) compared to all G3 patients with a median PFS1 of 7 months (Table 2).

**Figure 7:**
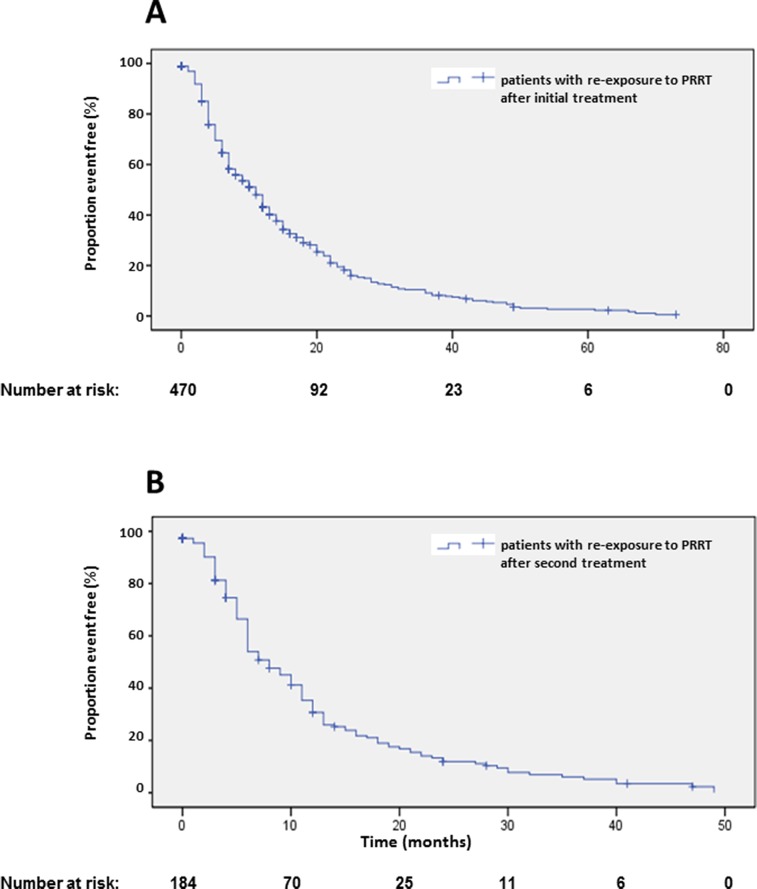
Kaplan-Meier plots of progression-free survival 2 **(A)** and 3 **(B)** after re-start of PRRT.

Origin of primary tumors influenced PFS. Patients with neuroendocrine neoplasias of small bowel had a longer PFS compared to patients with pancreatic NENs whereas bronchial origin und unknown origin was associated with a significantly shorter PFS in multi-variate analysis (Figure 6A, Table 2). The 25 patients with medullary thyroid cancer had a median PFS1 of 10.1 months (2-18.2). After an interval of more than 6 months, 470 patients underwent re-PRRT. Median PFS2 was 11 months (Figure 7A; Supplementary Table 1). Univariate analysis indicated statistically significant differences according to radionuclides, grading and origin of neuroendocrine neoplasia but no influence of gender, age, number of previous therapies and functionality (Supplementary Table 1). In multi-variate analysis, these differences were attributable to significantly lower PFS in patients treated with ^90^Y solely (Supplemantary Table 1). Patients with unknown grading had a significantly longer progression free survival with a median of 11 months, as compared to 9 months in patients with G2 tumors (Supplementary Figure 4A, Supplementary Table 1).

In 184 patients, a re-re-PRRT was performed after an interval of more than 6 months after re-PRRT (Supplementary Table 2). Median PFS was 8 months (Figure 7B). Here, the small size of the different groups limited the analysis of statistical significance. Uni-variate and multi-variate analysis suggested a significantly longer PFS in female patients (Supplementary Table 2). Multi-variate analysis indicated a shorter PFS in patients younger than 40 years and longer PFS in patients with unknown grading (Supplementary Figure 4B, Supplemenary Table 2), however, these results were limited by the small numbers of sub-groups.

### Adverse events

Adverse events were recorded according to patient and treatment cycle according to CTCAE criteria (Table 3). Hemoglobin levels, number of leucocytes, platelets and creatinine levels were determined before and after PRRT. After the initial PRRT (1048 cycles), grade 3 and 4 adverse events were rare. Dialysis was performed before PRRT in 3 patients and continued thereafter. After 2633 follow-up cycles, grade 3 and 4 adverse events were still prevalent in less than 1% of patients. Laboratory values were then studied across all 3692 cycles. Here, grade 3 and 4 adverse events did not increase and were still below 1%. Missing values were less than 1% (Table 3). Dialysis was necessary at any time after PRRT in additional 5 patients of 1048 (0.4%) patients. During follow-up, MDS or leukemia developed in 22 (2%) patients (Table 4), which developed after a mean of 8 years after diagnosis of the neuroendocrine neoplasia and was most prevalent in patients with NEN of pancreatic or small bowel. Possible risk factors included previous chemotherapy and external beam radiation. Most patients developing MDS or leukemia had a high tumor load with hepatic metastases. Prognosis of patients with MDS/leukemia was dismal with a mean overall survival of 14.4 months after diagnosis (Table 4).

**Table 3:** Number of patients with adverse events according to CTCAE criteria

Before first PRRT (1048)				
	Normal, G1, G2	G3	G4	No information
Leucocytes	1047 (99.9%)	0	0	1
Thrombocytes	1047 (99.9%)	0	3	1
Hemoglobin	1040 (99.2%)	3	3	1
Chronic kidney disease	1040 (99.2%)	1	2	4 (0.3%)
**According to treatment cycles (3692)**				
	**Normal, G1, G2**	**G3**	**G4**	**No information**
Leucocytes	3680 (99.6%)	8 (0.2%)	0	4 (0.1%)
Thrombocytes	3679 (99.6%)	1	8 (0.2%)	4 (0.1%)
Hemoglobin	3666 (99.2%)	10 (0.2%)	7	9 (0.2%)
Chronic kidney disease	3664 (99.2%)	7 (0.1%)	7 (0.2%)	14 (0.3)

**Table 4:** Characteristics of patients with MDS or leukemia after PRRT

	Age at diagnosis of neuroendocrine neoplasia	Age at start of PRRT	Age at diagnosis of MDS/leukemia	Radionuclides and dose	Type of MDS/leukemia	Type and extend of neuroendocrine neoplasia	Other risk factors	Sex	Survival after diagnosis of MDS/leukemia (death or date last seen)
1	40 years	42 years	49 years	^131^Iodine MIBG 22.7 GBq^90^Yttrium 7.4 GBq	MDS	paraganglioma hepatic, lymphatic and osseous metastases	chemotherapy external beam radiation (40 Gy)	female	15 months
2	67 years	68 years	69 years	^90^Yttrium 8 GBq^177^Lutetium 6 GBq	RAEB/T	neuroendocrine carcinoma pancreas hepatic, lymphatic and osseous metastases	right sided heart disease hypertension	female	6 months
3	66 years	79 years	80 years	^90^Yttrium 9.5 GBq ^177^Lutetium 6.3 GBq	RAEB-1	neuroendocrine tumor of unknown primary hepatic metastases	right sided heart disease hypertension	female	16 months
4	63 years	64 years	66 years	^90^Yttrium 14 GBq	RAEB/AML	neuroendocrine tumor pancreas extensive hepatic metastases	chemotherapy interferon alpha	male	8 months
5	66 years	67 years	69 years	^90^Yttrium 10.5 GBq ^177^Lutetium 4 GBq	acute leukemia	neuroendocrine carcinoma pancreas hepatic and lymphatic metastases		male	1 month
6	64 years	64 years	75 years	^177^Lutetium 8 GBq	RAEB-1	neuroendocrine tumor of the ileum lymphatic and hepatic metastases	external beam radiation (50 Gy)	female	11months
7	50 years	68 years	71 years	^90^Yttrium 2.5 GBq ^177^Lutetium 17 GBq	RAEB-1	neuroendocrine tumor of the ileum lymphatic and osseus metastases	arterial hypertension obesity	female	43 months
8	69 years	69 years	70 years	^90^Yttrium 11.4 GBq ^177^Lutetium 6.5 GBq	P-CLL	neuroendocrine tumor of unknown primary hepatic metastases	diabetes coronary heart disease arterial hypertension	male	96 months
9	63 years	63 years	67 years	^90^Yttrium 6.9 GBq ^177^Lutetium 27.3 GBq	MDS (CMML-1)	neuroendocrine tumor of the pancreas lymphatic and bone metastases	chemotherapy external beam radiation	male	8 months
10	69 years	70 years	70 years	^90^Yttrium 6 GBq	MDS RAEB-1	neuroendocrine tumor of the ileum extensive hepatic metastases		female	24 months
11	45 years	57 years	66 years	^90^Yttrium 16.6 GBq ^177^Lutetium 11 GBq	MDS RAEB-1	neuroendocrine carcinoma of the duodenum hepatic metastases	multiple chemotherapies Interferon alpha	female	16 months
12	51 years	52 years	58 years	^90^Yttrium 8 GBq ^177^Lutetium 22.5 GBq	MDS RAEB-1	pancreatic neuroendocrine neoplasma Lymphatic, osseus and extensive hepatic metastases	diabetes	male	8 months
13	69 years	70 years	71 years	^90^Yttrium 9.2 GBq ^177^Lutetium 12.7 GBq	t-MDS RAEB-2	pancreatic neuroendocrine neoplasma osseus and extensive hepatic metastases	chemotherapy	male	16 months
14	58 years	73 years	79 years	^177^Lutetium 22.5 GBq	AML MO	neuroendocrine neoplasma of ileum peritoneal and hepatic metastases	chemotherapy arterial hypertension	female	10 months
15	63 years	63 years	66 years	^177^Lutetium 26.3 GBq	MDS RAEB-1	neuroendocrine neoplasma of ileum Peritoneal, pleural, osseus and lymphatic metastases	external beam radiation carcinoid heart disease	female	6 months
16	49 years	52 years	57 years	^90^Yttrium 9.5 GBq ^177^Lutetium 11.7 GBq	MDS RAEB-1	pancreatic neuroendocrine neoplasma extensive lymphatic and hepatic metastases		female	16 months
17	71 years	73 years	75 years	^90^Yttrium 6.7 GBq ^177^Lutetium 6.9 GBq	AML EGIL	pancreatic neuroendocrine neoplasma extensive lymphatic, osseus and hepatic metastases	arterial hypertension diabetes mellitus	male	24 months
18	41 years	46 years	50 years	^90^Yttrium 8 GBq ^177^Lutetium 17.5 GBq	MDS RAEB-1	functionally active neuroendocrine neoplasm of ileum (carcinoid) extensive hepatic, bone, lymphatic and pleural metastases	external beam radiation diabetes mellitus	male	4 months
19	68 years	69 years	75 years	^90^Yttrium 5.2 GBq ^177^Lutetium 27.6 GBq	MDS RAEB-1	functionally active pancreatic neuroendocrine neoplasma (gastrinoma) lymphatic and extensive hepatic metastases	chemotherapy transarterial chemoembolisation molecular therapy	female	9 months
20	52 years	59 years	61 years	^177^Lutetium 21.7 GBq	MDS RAEB-2	typical carcinoid of the lung extensive hepatic and osseus metastases	selective internal radiotherapy external beam radiation hypertension	male	6 months
21	61 years	64 years	65 years	^177^Lutetium 8.5 GBq	MDS RAEB-1 AML	functionally active neuroendocrine neoplasm of ileum (carcinoid) extensive hepatic metastases	multiple TACE carcinoid heart disease arterial hypertension interferon therapy	male	16 months
22	53 years	53 years	63 years	^90^Yttrium 2 GBq ^177^Lutetium 48 GBq	MDS RAEB-1	pancreatic neuroendocrine neoplasma extensive lymphatic, osseous and hepatic metastases	chemotherapy	female	2 months
Ø	58.9 years	62.9 years	66.9 years	21.7 GBq	MDS unspecified 1 RAEB-1 12 RAEB-2 3 RAEB-T 1 AML 3 pCLL 1	Paraganglioma 1 SiNET 7 pNET (& duodenum) 9 CUP 2 Lung 1 Functionally active 2/21	ext. beam radiation 5 chemotherapy 8 carcinoid heart disease 4 interferon 3 art. hypertension 6 diabetes 3	12 female 10 male	14.4 months

Abbreviations: AML: acute myeloid leukemia; Ci: Curie; MDS: myelodysplastic syndrome; Gy: Gray; RAEB: refractory anemia with excess blasts; extensive hepatic metastases were defined as more than 50% infiltration of the liver data cut off for survival after diagnosis of MDS was 06-23-2016.

## DISCUSSION

In our study, we report OS, PFS and adverse events of 1048 patients treated by PRRT over ten years. Limitations of our study is it retrospective nature and lack of control arms and random selectin for therapies. On the other hand, this is a large selection of patients treated in day-today practice yielding results useful in clinical settings. Just more than half of the cohort has survived during follow-up yielding exact overall survival data of patients and sub-groups. To ensure that all deceased patients were included in the analysis, several tracking mechanisms were employed at our institution as described above. Compared to historical controls [11], OS observed in our study is favorable implying that PRRT is effective. On the other hand, OS observed in our study may be mainly related to better differentiation of NEN since we included only patients with somatostatin receptor-expressing NEN. However, there appears to be no survival advantage of patients with somatostatin receptor-expressing mid-gut neuroendocrine tumors compared to non-expressing tumors [18].

Treatment with both radioisotopes resulted in better OS than with ^177^Lu or ^90^Y solely. These results were also reported in another study. Combined application of ^90^Y- and ^177^Lu-based PRRT in patients with different NENs resulted in a median OS of 66.1 months [19], which is almost identical to the median OS of 64 months in our study. These results indicate that personalized treatment adapting radioisotopes and dose to tumor load and location is superior than more restricted protocols. However, it should be noted that PRRT as monotherapy may be not as effective as combinatory protocols using radiosensitizers such as Everolimus (Afinitor®) or a combination of capecitabine and temozolomide achieving higher response rates compared to PRRT alone [20, 21].

Another molecular target, the serine-threonine kinase mTOR plays a pivotal role in pathogenesis of NENs and is effectively inhibited by Everolimus (Afinitor®). A prospective placebo-controlled multi-center trial in patients with pancreatic NENs has shown prolongation of PFS in patients treated with Everolimus (Afinitor®) versus patients treated with placebo (11.0 versus 4.6 months). Although median PFS in patients treated with Everolimus (Afinitor®) is lower in patients with pancreatic NENs than 20 months observed in our study, a recent analysis has shown that patients treated initially with Everolimus (Afinitor®) have a median OS of 44 months [22], which corresponds exactly to the median OS in our study. Albeit these studies are difficult to compare due to different design, these results corroborated clinical significance of hitting key molecular pathways in treatment of NENs.

OS was significantly determined by gender, age, radionuclides, number of previous therapies and origin of tumors in uni- and multi-variate analysis whereas functionality of NENs was significantly different only in uni-variate but not in multi-variate analysis. The patients with G1 tumors had the best OS, and so also did those with an age of a less than 40 years at diagnosis, patients treated with a combination of ^90^Y and ^177^Lu, and in NENs of small bowel. The OS of patients with G3 NENs was favorable. However, it is likely that this subgroup reflects mainly well differentiated neuroendocrine carcinomas due to the expression of somatostatin receptors. This group most likely reflects the newly created classification of neuroendocrine tumor G3, which is limited to NENs with a proliferation rate of up to 50% [3]. Indeed, OS of patients with a proliferation rate of more than 50% had a shorter OS than the whole group. These differences reflect biological characteristics of NENS, which should be included in clinical practice when planning PRRT and follow-up.

Follow-up in our patients was performed with ^68^Ga-SSTR PET/CT and EORTC criteria as well as RECIST [23]. Median PFS was 19 months in all patients, which is considerably shorter than the median of 41 months determined by RECIST 1.1 in a multi-institutional study in Germany involving 450 patients [11]. Shorter PFS by SSTR PET/CT follow-up reflects the increased sensitivity of this novel method. Although, ^68^Ga-SSTR PET/CT is very accurate for estimating the therapy response and especially for patient selection for PRRT, we postulate that PFS calculated solely on the basis of PET/CT be judged with caution. It should be kept in mind that EORTC criteria were primarily meant for the assessment of tumor response in lymphomas on ^18^F-FDG PET/CT. The different principle of assessment (SSTR status vs glucose metabolism) of NENs by this molecular imaging technique has to be born in mind. Probably, there is a need for newer response assessment criteria (for e.g., based on SUV tumor-to-spleen ratio, molecular tumor volume), designed on the basis of future prospective studies. In fact, median PFS determined by SSTR PET/CT using EORTC criteria does not mandatorily mirror OS in sub-groups with divergent prognosis. In fact, median PFS was not significantly different according to gender and age. Overall survival advantage of patient treated with a combination of ^90^Y and ^177^Lu (64 months) compared to sole usage of ^177^Lutetium (44 months) was not exactly reflected in median PFS times of 24 and 17 months, respectively, although showing a similar trend. Only exclusive usage of ^90^Y as radionuclide for PRRT resulted in significant lower PFS of 7 months compared to combined use (24 months). Likewise, OS advantage of patients with G1 NEN compared to G2 NEN (88 months versus 55 months) was not translated into PFS, which was 22 months for G1 tumors and 21 months for G2 NENs. However, G3 NENs had a lower OS and PFS. Number of previous therapies and functionality had no significant effect upon median PFS in multi-variate analysis. Median PFS in NENs of bronchial or unknown primary was significantly lower compared to pancreatic NENs in multi-variate analysis. Interestingly, the significantly lower median PFS of NENs of unknown primary (13 months versus 20 months in pancreatic NENs) was not reflected in median OS, which was not significantly different in both sub-groups (53 versus 44 months). We were able to include a subgroup of patients with somatostatin receptor positive medullary thyroid cancer in our analysis. Median OS of these patients was promising with 32 months median OS and 10.1 months PFS1, which should be examined in larger studies comparing standard treatments and peptide receptor radionuclide therapy.

These results indicate that progression documented by a sensitive method does not necessarily affect OS and should be considered in clinical practice. A criteria/scoring for somatostatin receptor PET/CT in NETs including for molecular response assessment, similar to for e.g., Krenning's score for somatostatin receptor scintigraphy, needs to be developed and standardized.

Patients with NENs often require multi-disciplinary treatment with several lines of systemic therapy, often resulting in side effects depending on the therapy involved. Hemo- and nephrotoxicity are well known adverse events of PRRT [11, 24–27]. We monitored adverse events in our study by screening laboratory measurements during the 3692 treatment cycles. Here, we found grade 3 and 4 adverse events in less than 1% of PRRT cycles. There could be a possible underestimation of the real extent, since long term effects may be potentially missed. Therefore, we performed an additional search in all patient files and also obtained extensive information from the family physician to look into any hematological and nephrological toxicities during follow-up. We identified 22 subjects with leukemia/MDS and 5 additional patients (0.4%) with chronic kidney disease requiring hemodialysis after PRRT, whereas 3 patients had already been on hemodialysis before PRRT due to other reasons. Recently, incidence of leukemia and MDS after PRRT was reviewed by Bodei et al. [26] and Kesavan & Turner [25] reporting rates of 2% and 1.4%, respectively which corresponds to the 2.1% in our study. Risk factors identified in our study were chemotherapy and external beam radiation, as well as high tumor loads defined as involvement of more than 50% of the liver, multiple/multifocal (<10) bone and lymph node metatases. Development of leukemia/MDS after PRRT is associated with a limited mean overall survival of 14.4 months in our study. Rates of permanent severe nephrotoxicity of grade ≥4 have been described in as high as 9.2% in a study applying high doses of ^90^Y exclusively [24]. In contrast, severe nephrotoxicity is rare in patients treated by PRRT with ^177^Lu [28]. Incidence of severe renal impairments in our study was much lower, due to the mandatory renal protection followed as well as proper hydration of the patient after therapy.

## MATERIALS AND METHODS

### Patients

Between 2004 and 2014, 2294 patients were referred to Zentralklinik Bad Berka, Germany for diagnosis and treatment of NENs. Of these, 570 were found to be not eligible for PRRT and 676 patients, although eligible, did not undergo the treatment for various reasons (e.g. decision was reached in the interdisciplinary tumor board to perform first a different kind of therapy like transcatheter arterial chemoembolisation due to high functionality of the tumor). The criteria used to define eligibility for PRRT were in conformation with the published guidelines for PRRT [29] and included a Karnofsky of more than 60%, life expectancy of more than 6 months, somatostatin receptor positive NENs and adequate renal and bone marrow function. Excluded were patients not matching these criteria and somatostatin receptor negative NENs. Thus, PRRT was performed in 1048 patients and all of them were included in the intention to treat analysis (Figure 1). Before PRRT, patients were extensively informed about the procedure and the possible side effects. Written informed consent was obtained from all patients, which included permission for data storage and analysis. In 2007, the responsible ethical committee permitted retrospective and prospective data collection and analysis by the German neuroendocrine tumor registry, which was renewed in 2014. Data collection was performed in accordance with the registry. Decision to treat the patients by PRRT was taken by internal or external tumor boards. All patients were studied by SSTR PET/CT using ^68^Ga-DOTANOC, ^68^Ga-DOTATOC, or ^68^Ga-DOTATATE prior to PRRT, which was also used for patient follow-up. All patients were either progressive before PRRT, as determined by morphological imaging (CT or MRI) or by SSTR PET/CT, or were severely symptomatic due to extensive tumor mass or functional syndromes.

### Radiopharmaceuticals

The DOTA-conjugated somatostatin analogues DOTA-TOC,-NOC and -TATE were labeled with ^68^Ga, ^177^Lu and ^90^Y in our radiopharmacy department in accordance with good medical practice. The radionuclide ^68^Ga was obtained in house from the ^68^Geranium-^68^Ga generator (Eckert and Ziegler GmbH, Berlin, Germany). A highly efficient NaCl-based labeling procedure for the radiopharmaceutical production of ^68^Ga labeled ligand-conjugate has been developed in our hospital [30, 31]. ^177^Lu and ^90^Y were obtained from different manufacturers. The labeling of DOTA-conjugated peptides with ^177^Lu (also applicable to ^90^Y) was performed according to a previously published method [32].

### ^68^Ga-SSTR PET/CT

PET/CT (until January 2014 with Siemens Biograph and since then with Biograph mCT Flow 64; Siemens Medical Solutions AG, Erlangen, Germany) was performed in all cases 45-90 minutes after the intravenous injection of 46-260 MBq of ^68^Ga-DOTANOC, -DOTATOC or DOTATATE. PET/CT images were acquired from the skull to the middle part of the thigh. Contrast-enhanced CT (ceCT, spiral CT using Biograph mCT Flow 64) was acquired after intravenous administration of 60-100 mL nonionic iodinated contrast. Images were evaluated by two experienced nuclear medicine specialists. Any area with intensity greater than background and that could not be explained by physiologic activity was considered to be indicative of tumor tissue. Maximum standardized uptake values (SUV_max_) were obtained by drawing circular regions of interest (ROIs), which were automatically adapted (40% isocontour) to a 3D volume of interest using commercial software provided by the vendor.

### Infusion and renal protection

Kidney protection was performed using an infusion of 1600 mL of a renoprotective amino-acid mixture of 5% lysine HCl and 10% L-arginine HCl. The infusion was started at least 30 min prior to administration of the therapeutic dose and continued for 4 h thereafter. The radiopharmaceutical was co-administered over 10–15 min by using a second infusion pump system. The activity to administer was individually chosen based on the uptake in the tumor lesions as shown by ^68^Ga-SSTR PET/CT (performed before each treatment cycle), kidney function (assessed using the following: tubular extraction rate determined by ^99m^Tc-MAG3 scintigraphy, glomerular filtration determined by ^99m^Tc-DTPA clearance, and serum creatinine), hematological reserve, previous treatments and general status of the patient (Karnofsky Performance Scale) [32–34]. Decision to use ^90^Y and/or ^177^Lu depended upon tumor mass, SUV and radionuclide availability [24]. Both radionuclides were used in subsets of patients sequentially (DUO) or in combination (TANDEM)[19, 35].

### PRRT

The intention to treat analysis included 1048 patients, who received 3692 cycles of PRRT. Most patients received a combination of ^90^Y and ^177^Lu (49%), whereas 36% patients were only treated with ^177^Lu and 15% with ^90^Y, exclusively. PRRT with ^90^Y was performed in 371 cycles, applying a mean activity of 3.18 GBq, whereas with ^177^Lu was administered solely in 1043 cycles with a mean applied activity of 6.54 GBq. Dosimetry was performed after each cycle. Administered activity depended on patient and tumor-related factors like weight, renal function, tumor burden, clinical course and previous dosimetry. DUO-PRRT, i.e., application of ^177^Lu and ^90^Y in subsequent cycles in a particular patient, comprised 1235 cycles with ^177^Lu (mean administered activity 6.55 GBq) and 1008 cycles with ^90^Y (mean activity 3.36 GBq). TANDEM-PRRT, i.e., treatment with a combination of ^177^Lu and ^90^Y-labeled somatostatin analogues administered on the same day, was performed in 35 cycles with a mean implemented activity of 3.06 and 4.65 GBq, respectively.

### Efficacy and safety

Restaging was performed with SSTR-PET/CT 3-6 months after PRRT. In case of stable disease or remission (complete or partial), the patient was restaged with SSTR PET/CT every 6-12 months until disease progression was evident on imaging. In addition, MRI in selected cases (allergy to iodinated contrast or poor detectability of liver metastases on CT scan) and routine sonography were performed for additional diagnostic evaluation. Resumption of PRRT after detection of progression after a therapy interruption of more than 6 months (2^nd^ phase of PRRT) was performed in 470 patients. The next (second) resumption of PRRT (3^rd^ phase of PRRT) after progression post 2^nd^ phase of PRRT after an additional interval of more than 6 months was implemented in 184 patients. Laboratory parameters (erythrocytes, haemoglobin, platelets, leucocytes, creatinine, BUN, SGOT, SGPT, bilirubin, SAP, TSH, SGamma-GT and respective tumor markers) were evaluated prior to each cycle and at restaging. Renal function was monitored by tubular extraction rate (TER) using ^99m^Tc-MAG3 renography and in addition the glomerular filtration rate (GFR) was determined using ^99m^Tc-DTPA renography.

### Method of patient documentation

A database was established in 2004 including more than 250 items for all patients with at least one cycle of PRRT. Out of this database, items were extracted to allow analysis of overall and progression-free survival according to age, sex, number and kind of pretreatments, grading, differentiation, methods of PRRT and administered radioactivity as well as origin of primary tumors and presence and kind of functional syndromes. Adverse events were determined according to CTCAE criteria version 4.03 by assessing all available laboratory measurements. In addition, adverse events were documented in an additional dataset by incoming patient reports during follow-up. Clinical data from the patient file as well as during patient registration by trained physician assistants. Data analysis was performed on anonymized data sets. All patients irrespective of tumor stage or prognosis, received an appointment for follow-up. When patients did not appear, their family and/or physicians were contacted. In addition, all patients were invited once yearly to a patient conference organized by our hospital. Date of death was reported by family physicians. No patient was lost to follow-up.

### Statistical analysis

We assessed overall survival and progression-free survival with Kaplan-Meier curves and compared subgroups with one-sided log rank test. Univariate and multivariate analyses were done in R version 3.0.0 and rms package [36]. Uni-variate analysis was done by fitting an analysis of variance model to each variable under investigation [37]. Multi-variate analysis was conducted using Cox models [38]. Responses were evaluated with EORTC criteria (PET component of PET/CT) as well as by RECIST (CT component of PET/CT or MRI) [39]. Adverse events were assessed from lab data and graded according to CTCAE criteria.

## CONCLUSIONS

Treatment of patients with NENs by PRRT has demonstrated favorable and clinical significant median OS and PFS with minimal acceptable side effects, observed during a treatment period of a decade. Follow-up by somatostatin receptor PET/CT is feasible and effective, however, progression documented by this sensitive method should be always evaluated in clinical context.

## SUPPLEMENTARY MATERIALS FIGURES AND TABLES



## Abbreviations

^177^Lu: ^177^Lutetium
^68^Ga: ^68^Gallium
^68^Ga-DOTANOC: ^68^Gallium [DOTA, 1-Nal3]-octreotide (DOTANOC)
^68^Ga-DOTATATE: ^68^Gallium [DOTA, Tyr3]-octreotate
^90^Y: ^90^Yttrium
^99m^Tc-DTPA: Technetium-99mTc-diethylenetriaminepentaacetic acid
^99m^Tc-MAG3: Technetium-99m (99mTc) mercaptoacetyltriglycine
Bq: Bequerel
BUN: blood urea nitrogen
CTCAE: Common Terminology Criteria for Adverse Events
DOTATOC: (DOTA(0)-Phe(1)-Tyr(3))octreotid
EORTC: European Organisation for Research and Treatment of Cancer
MRI: magnetic resonance imaging
NEN: neuroendocrine neoplasms
OS: overall survival
PET/CT: positron-emission tomography/computed tomography
PFS: progression-free survival
PRRT: peptide receptor radionuclide therapy
SAP: serum alkaline phosphatase
SGamma-GT: serum γ-Glutamyltransferase
SGOT: serum glutamic-oxaloacetic transaminase
SGPT: glutamic-pyruvic transaminase
SSTR: somatostatin receptor
SUV: standardized uptake values
TER: tubular extraction rate
TSH: tyroidea stimulating hormone
WHO: world health organization

## References

[1] Merola E, Rinzivillo M, Cicchese N, Capurso G, Panzuto F, Delle Fave G (2016). Digestive neuroendocrine neoplasms: A 2016 overview. Dig Liver Dis.

[2] Rindi G, Petrone G, Inzani F (2014). The 2010 WHO classification of digestive neuroendocrine neoplasms: a critical appraisal four years after its introduction. Endocr Pathol.

[3] Klöppel G (2017). Neuroendocrine Neoplasms: Dichotomy, Origin and Classifications. Visc Med.

[4] de Herder WW, Rehfeld JF, Kidd M, Modlin IM (2016). A short history of neuroendocrine tumours and their peptide hormones. Best Pract Res Clin Endocrinol Metab.

[5] Rinke A, Krug S (2016). Neuroendocrine tumours - Medical therapy: Biological. Best Pract Res Clin Endocrinol Metab.

[6] Rinke A, Wittenberg M, Schade-Brittinger C, Aminossadati B, Ronicke E, Gress TM, Müller HH, Arnold R, PROMID Study Group (2017). Placebo-Controlled, Double-Blind, Prospective, Randomized Study on the Effect of Octreotide LAR in the Control of Tumor Growth in Patients with Metastatic Neuroendocrine Midgut Tumors (PROMID): Results of Long-Term Survival. Neuroendocrinology.

[7] Caplin ME, Pavel M, Ćwikła JB, Phan AT, Raderer M, Sedláčková E, Cadiot G, Wolin EM, Capdevila J, Wall L, Rindi G, Langley A, Martinez S, CLARINET Investigators (2016). Anti-tumour effects of lanreotide for pancreatic and intestinal neuroendocrine tumours: the CLARINET open-label extension study. Endocr Relat Cancer.

[8] Deppen SA, Liu E, Blume JD, Clanton J, Shi C, Jones-Jackson LB, Lakhani V, Baum RP, Berlin J, Smith GT, Graham M, Sandler MP, Delbeke D (2016). Safety and Efficacy of 68Ga-DOTATATE PET/CT for Diagnosis, Staging, and Treatment Management of Neuroendocrine Tumors. J Nucl Med.

[9] Bodei L, Kwekkeboom DJ, Kidd M, Modlin IM, Krenning EP (2016). Radiolabeled Somatostatin Analogue Therapy Of Gastroenteropancreatic Cancer. Semin Nucl Med.

[10] Brabander T, Teunissen JJ, Van Eijck CH, Franssen GJ, Feelders RA, de Herder WW, Kwekkeboom DJ (2016). PRRT of neuroendocrine tumours. Best Pract Res Clin Endocrinol Metab.

[11] Hörsch D, Ezziddin S, Haug A, Gratz KF, Dunkelmann S, Miederer M, Schreckenberger M, Krause BJ, Bengel FM, Bartenstein P, Biersack HJ, Pöpperl G, Baum RP (2016). Effectiveness and side-effects of PRRT for neuroendocrine neoplasms in Germany: A multi-institutional registry study with prospective follow-up. Eur J Cancer.

[12] Strosberg J, El-Haddad G, Wolin E, Hendifar A, Yao J, Chasen B, Mittra E, Kunz PL, Kulke MH, Jacene H, Bushnell D, O'Dorisio TM, Baum RP (2017). Phase 3 trial of 177Lu-Dotatate for midgut neuroendocrine tumors. N Engl J Med.

[13] Haug AR, Auernhammer CJ, Wängler B, Schmidt GP, Uebleis C, Göke B, Cumming P, Bartenstein P, Tiling R, Hacker M (2010). 68Ga-DOTATATE PET/CT for the early prediction of response to somatostatin receptor-mediated radionuclide therapy in patients with well-differentiated neuroendocrine tumors. J Nucl Med.

[14] Ezziddin S, Lohmar J, Yong-Hing CJ, Sabet A, Ahmadzadehfar H, Kukuk G, Biersack HJ, Guhlke S, Reichmann K (2012). Does the pretherapeutic tumor SUV in 68Ga DOTATOC PET predict the absorbed dose of 177Lu octreotate?. Clin Nucl Med.

[15] Sainz-Esteban A, Prasad V, Schuchardt C, Zachert C, Carril JM, Baum RP (2012). Comparison of sequential planar 177Lu-DOTA-TATE dosimetry scans with 68Ga-DOTA-TATE PET/CT images in patients with metastasized neuroendocrine tumours undergoing PRRT. Eur J Nucl Med Mol Imaging.

[16] Kratochwil C, Stefanova M, Mavriopoulou E, Holland-Letz T, Dimitrakopoulou-Strauss A, Afshar-Oromieh A, Mier W, Haberkorn U, Giesel FL (2015). SUV of [68Ga]DOTATOC-PET/CT Predicts Response Probability of PRRT in Neuroendocrine Tumors. Mol Imaging Biol.

[17] Baum RP, Kluge AW, Kulkarni H, Schorr-Neufing U, Niepsch K, Bitterlich N, van Echteld CJ (2017). [(177)Lu-DOTA](0)-D-Phe(1)-Tyr(3)-Octreotide ((177)Lu-DOTATOC) For Peptide Receptor Radiotherapy in Patients with Advanced Neuroendocrine Tumours: A Phase-II Study. Theranostics.

[18] Wang YZ, Beyer DT (2016). Does receptor status impact survival of patients with mid-gut neuroendocrine tumors. J Clin Oncol.

[19] Villard L, Romer A, Marincek N, Brunner P, Koller MT, Schindler C, Ng QK, Mäcke HR, Müller-Brand J, Rochlitz C, Briel M, Walter MA (2012). Cohort study of somatostatin-based radiopeptide therapy with [(90)Y-DOTA]-TOC versus [(90)Y-DOTA]-TOC plus [(177)Lu-DOTA]-TOC in neuroendocrine cancers. J Clin Oncol.

[20] Claringbold PG, Turner JH (2015). NeuroEndocrine Tumor Therapy with Lutetium-177-octreotate and Everolimus (NETTLE): A Phase I Study. Cancer Biother Radiopharm.

[21] Claringbold PG, Turner JH (2016). Pancreatic Neuroendocrine Tumor Control: Durable Objective Response to Combination 177Lu-Octreotate-Capecitabine-Temozolomide Radiopeptide Chemotherapy. Neuroendocrinology.

[22] Yao JC, Pavel M, Lombard-Bohas C, Van Cutsem E, Lam D, Kunz T, Brandt U, Capdevila J, De Vries EGE, Tomassetti P, Hobday T, Pommier R, Öberg K (2016). Everolimus for the Treatment of Advanced Pancreatic Neuroendocrine Tumors: Overall Survival and Circulating Biomarkers From the Randomized, Phase III RADIANT-3 Study. J Clin Oncol.

[23] Aras M, Erdil TY, Dane F, Gungor S, Ones T, Dede F, Inanir S, Turoglu HT (2016). Comparison of WHO, RECIST 1.1, EORTC, and PERCIST criteria in the evaluation of treatment response in malignant solid tumors. Nucl Med Commun.

[24] Imhof A, Brunner P, Marincek N, Briel M, Schindler C, Rasch H, Mäcke HR, Rochlitz C, Müller-Brand J, Walter MA (2011). Response, survival, and long-term toxicity after therapy with the radiolabeled somatostatin analogue [90Y-DOTA]-TOC in metastasized neuroendocrine cancers. J Clin Oncol.

[25] Kesavan M, Turner JH (2016). Myelotoxicity of PRRT of Neuroendocrine Tumors: A Decade of Experience. Cancer Biother Radiopharm.

[26] Bodei L, Modlin IM, Luster M, Forrer F, Cremonesi M, Hicks RJ, Ezziddin S, Kidd M, Chiti A (2016). Myeloid neoplasms after chemotherapy and PRRT: myth and reality. Endocr Relat Cancer.

[27] Sabet A, Ezziddin K, Pape UF, Ahmadzadehfar H, Mayer K, Pöppel T, Guhlke S, Biersack HJ, Ezziddin S (2013). Long-term hematotoxicity after PRRT with 177Lu-octreotate. J Nucl Med.

[28] Bergsma H, Konijnenberg MW, van der Zwan WA, Kam BL, Teunissen JJ, Kooij PP, Mauff KA, Krenning EP, Kwekkeboom DJ (2016). Nephrotoxicity after PRRT with (177)Lu-DOTA-octreotate. Eur J Nucl Med Mol Imaging.

[29] Bodei L, Mueller-Brand J, Baum RP, Pavel ME, Hörsch D, O'Dorisio MS, O'Dorisio TM, Howe JR, Cremonesi M, Kwekkeboom DJ (2013). The joint IAEA, EANM, and SNMMI practical guidance on PRRT (PRRNT) in neuroendocrine tumours. Eur J Nucl Med Mol Imaging.

[30] Mueller D, Klette I, Baum RP, Gottschaldt M, Schultz MK, Breeman WA (2012). Simplified NaCl based (68)Ga concentration and labeling procedure for rapid synthesis of (68)Ga radiopharmaceuticals in high radiochemical purity. Bioconjug Chem.

[31] Mueller D, Breeman WAP, Klette I, Gottschaldt M, Odparlik A, Baehre M, Schultz MK (2016). Radiolabeling of DOTA-like conjugated peptides with generator-produced Ga-68 and using NaCl-based cationic elution method. Nat Protoc.

[32] Wehrmann C, Senftleben S, Zachert C, Müller D, Baum RP (2007). Results of individual patient dosimetry in PRRT with 177Lu DOTA-TATE and 177Lu DOTA-NOC. Cancer Biother Radiopharm.

[33] Kulkarni HR, Schuchardt C, Baum RP (2013). PRRT with (177)Lu labeled somatostatin analogs DOTATATE and DOTATOC: contrasting renal dosimetry in the same patient. Recent Results Cancer Res.

[34] Schuchardt C, Kulkarni HR, Prasad V, Zachert C, Müller D, Baum RP The Bad Berka dose protocol: comparative results of dosimetry in PRRT using (177)Lu-DOTATATE, (177)Lu-DOTANOC, and (177)Lu-DOTATOC. Recent Results Cancer Res.

[35] Baum RP, Kulkarni HR, Carreras C (2012). Peptides and receptors in image-guided therapy: theranostics for neuroendocrine neoplasms. Semin Nucl Med.

[36] R Core Team (2012). R: A language and environment for statistical computing. R foundation for Statistical Computing, Vienna, Austria.

[37] Chambers JM, Freeny AE, Heiberger RM (1992). Analysis of variance; designed experiments. In Statistical Models Eds JM Chambers & TJ Hastie.

[38] Cox DR (1972). Regression models and life tables (with discussion). Journal of the Royal Statistical Society, Series B.

[39] Young H, Baum R, Cremerius U, Herholz K, Hoekstra O, Lammertsma AA, Pruim J, Price P (1999). Measurement of clinical and subclinical tumour response using [18F]-fluorodeoxyglucose and positron emission tomography: review and 1999 EORTC recommendations. European Organization for Research and Treatment of Cancer (EORTC) PET Study Group. Eur J Cancer.

