# Supplementary material for: Results and adverse events of personalized peptide receptor radionuclide therapy with 90Yttrium and 177Lutetium in 1048 patients with neuroendocrine neoplasms
Source: Oncotarget. 2018 Feb 15;9(24):16932–50. doi: 10.18632/oncotarget.24524 (PMC5908296; doi:10.18632/oncotarget.24524)
Supplement: Supplementary file 1 [file oncotarget-09-16932-s001.pdf]

Results and adverse events of personalized peptide receptor radionuclide therapy with ^{90}Yttrium and ^{177}Lutetium in 1048 patients with neuroendocrine neoplasms

SUPPLEMENTARY MATERIALS

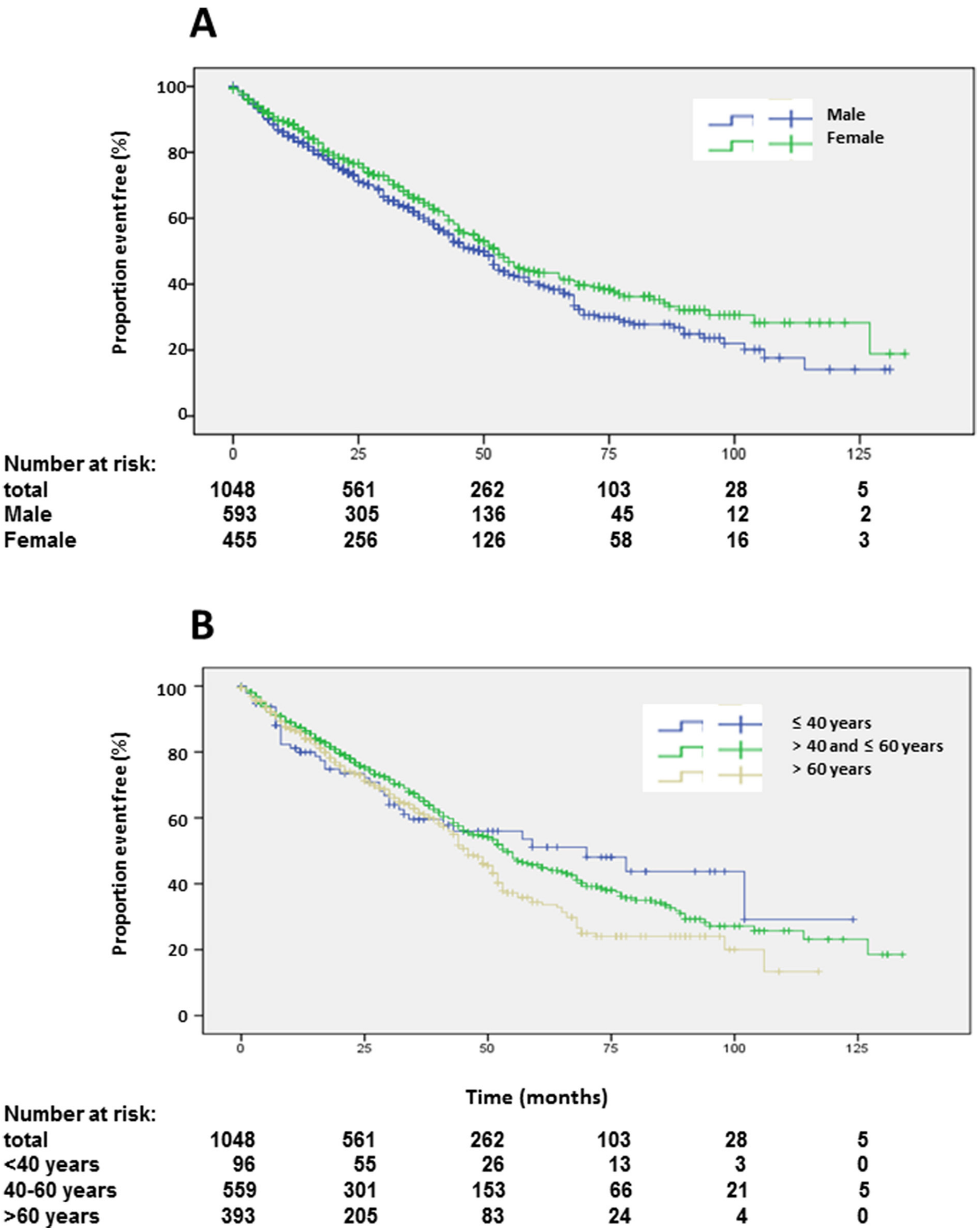

Supplementary Figure 1: Kaplan-Meier plots of overall survival according to gender **(A)** and age **(B)**.

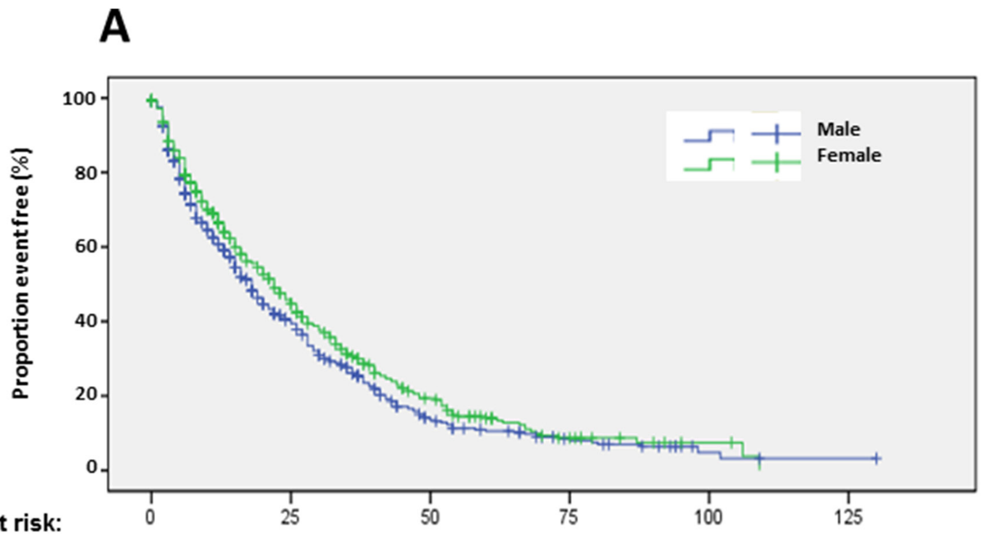

Number at risk:						
total	1048	340	94	30	6	1
Male	593	179	45	17	3	1
Female	455	161	49	13	3	0

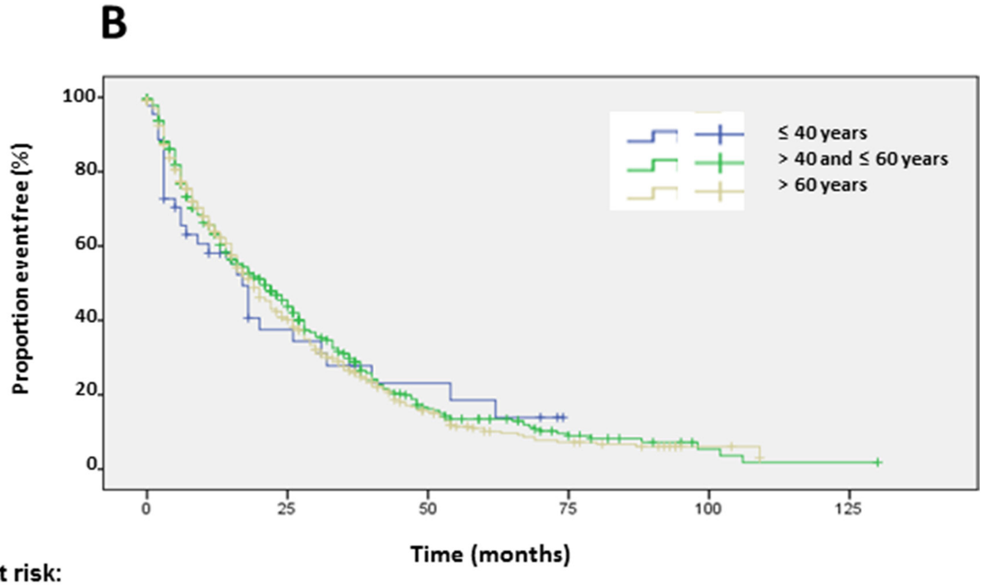

Number at risk:						
total	1048	340	94	30	6	1
<40 years	96	33	12	4	1	0
40-60 years	559	190	48	16	4	1
>60 years	393	117	34	10	1	0

Supplementary Figure 2: Kaplan-Meier plots of progression-free survival 1 according to gender **(A)** and age **(B)**.

A

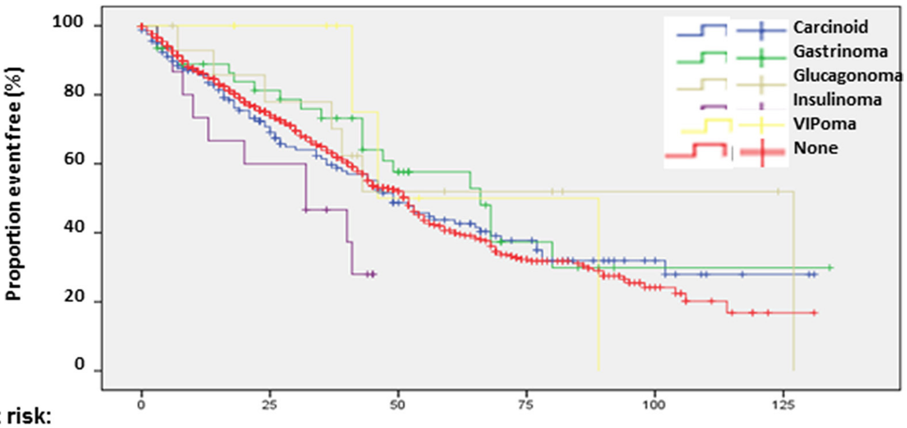

Number at risk:						
Total	1048	561	262	103	28	5
Carcinoid Syn.	158	87	50	28	9	2
Gastrinoma	46	31	18	5	1	1
Glucagonoma	16	10	5	4	2	1
Insulinoma	15	9	0	0	0	0
VIPoma	8	6	2	1	0	0
None	804	418	187	65	16	1

B

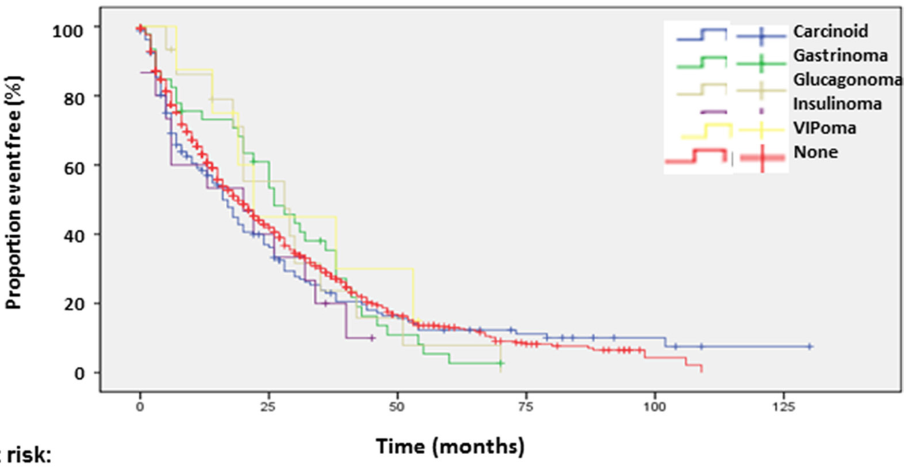

Number at risk:						
Total	1048	340	94	30	6	1
Carcinoid Syn.	158	50	20	10	4	1
Gastrinoma	46	24	4	0	0	0
Glucagonoma	16	7	2	0	0	0
Insulinoma	15	6	0	0	0	0
VIPoma	8	3	2	0	0	0
None	804	250	66	20	2	0

Supplementary Figure 3: Kaplan-Meier plots of overall survival **(A)** and progression-free survival **(B)** according to functionality. Abbreviations: Carcinoid Syn.: Carcinoid syndrome.

A

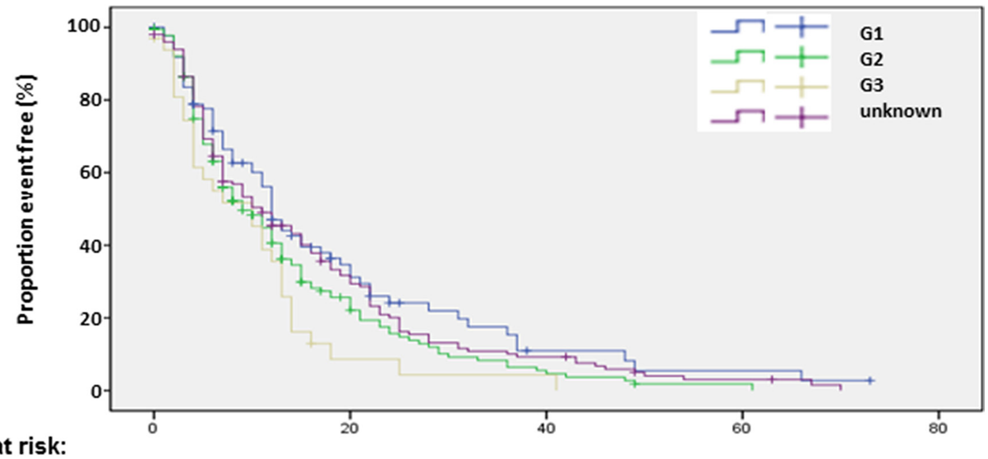

Number at risk:					
total	470	92	23	6	0
G1 (<=2)	96	20	4	2	0
G2 (3-20)	190	29	6	1	0
G3 (>20)	33	2	1	0	0
unknown	151	41	12	3	0

B

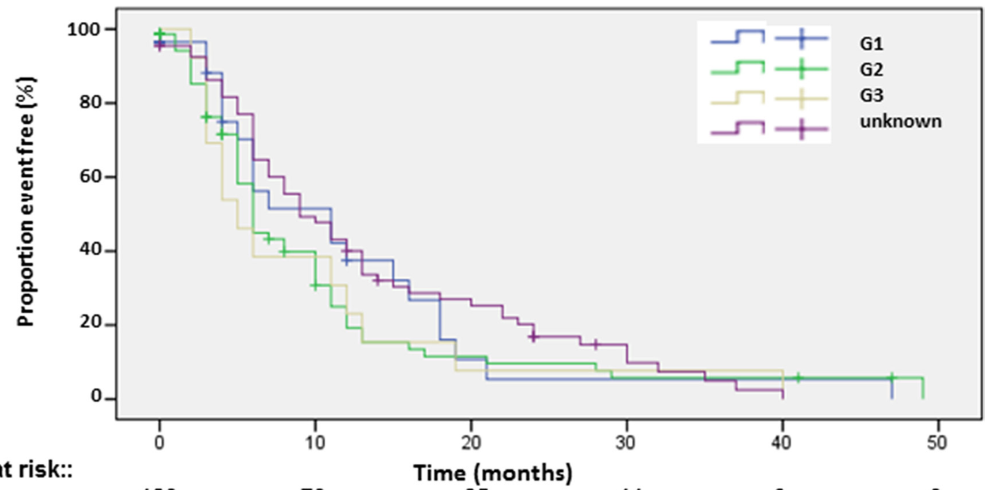

Number at risk::						
Total	180	70	25	11	6	0
G1 (<=2)	29	11	2	1	1	0
G2 (3-20)	75	22	6	3	3	0
G3 (>20)	13	5	1	1	1	0
unknown	67	32	16	6	1	0

Supplementary Figure 4: Kaplan-Meier plots of progression-free survival 2 **(A)** and 3 **(B)** according to grading.

Supplementary Table 1: Patient characteristics and results of progression-free survival survival 2 after first re-uptake of PRRT

analysis	Number	%	Progressive patients	Median	95% CI	Univariate analysis		Multivariate analysis	
						p	HR	95% CI	p
**All Patients**	470	100	376	11	9.4-12.5				
**Gender**									
Male	277	59	220	11	8.7-13.2	0.224	1		
Female	193	41	156	11	9.3-12.6		0.95	0.77-1.18	0.66
**Age**									
≤40 years	43	9.1	31	8	3.8-12.1	0.349	0.99	0.66-1.49	0.97
>40 and ≤60 years	253	53.8	199	12	10.1-13.9		1		
>60 years	174	37.1	146	10	7.6-12.3		1.19	0.95-1.5	0.14
**Radionuclide**									
Lutetium-177	116	24.6	83	11	8.1-13.8	**0.0085**	1.12	0.86-1.45	0.4
Yttrium-90	39	8.2	36	5	3.8-6.1		2.01	1.36-2.96	**0.0004**
Combined	257	54.6	257	12	10.2-13.7		1		
**Grading**									
G1 (Ki67<2%)	96	20.4	66	12	10.1-13.8	**0.0144**	0.82	0.61-1.11	0.21
G2 (Ki67 3-20%)	190	40.4	145	9	6.2-11.7		1		
G3 (Ki67>20%)	33	7	30	10	4.5-15.4		1.09	0.71-1.66	0.72
Unknown	151	32.2	135	11	7.4-14.5		0.78	0.61-0.99	**0.04**
**Previous therapies (other than first course of PRRT)**									
0	26	5.5	17	12	5.1-18.8	0.566	0.79	0.47-1.33	0.38
1	149	31.7	114	12	10.1-13.8		0.90	0.7-1.17	0.45
2-3	198	42.1	165	18	6.9-11		1		
>3	97	20.6	80	17	6.4-13.5		1.01	0.76-1.36	0.93
**Primary tumor**									
Bronchial	43	9.2	35	8	6-9.9	**0.0251**	0.95	0.64-1.41	0.83
Pancreas	193	41	160	10	7.7-12.2		1		
Small Intestine	117	24.9	88	12	9.2-14.7		0.62	0.46-0.85	**0.0025**
CUP	65	13.9	50	11	7.1-14.8		0.95	0.67-1.36	0.81
Other	52	11	43	11	6.9-15		0.95	0.67-1.35	0.79
**Functional syndromes**									
Carcinoid syndrome	72	15.3	61	12	7.3-16.6	0.182	0.99	0.72-1.34	0.93
Gastrinoma	27	5.7	17	13	9.4-16.5		0.72	0.44-1.21	0.22
Insulinoma	9	1.9	8	5	1-8.9		1.78	0.86-3.72	0.12
Glucagonoma	9	1.9	8	6	nr-12.4		0.76	0.37-1.59	0.48
VIPoma	6	1.2	5	10	8.3-11.6		1.3	0.51-3.33	0.59
None	347	73.8	277	11	9.4-12.5		1		

Abbreviations: nr: not reached; 95 CI: 95% confidence interval bold figures indicate significance.

Supplementary Table 2: Patient characteristics and results of progression-free survival 3 after second re-uptake of PRRT

analysis	Number	%	Progressive patients	Median	95% CI	Univariate analysis		Multivariate analysis	
						p	HR	95% CI	p
**All Patients**	184	100	151	8	6.4-9.5				
**Gender**									
Male	114	62	90	7	5.4-8.5	**0.0057**	1		
Female	70	38	61	11	7.8-14.1		0.66	0.45-0.96	**0.03**
**Age**									
≤40 years	8	4.3	6	4	1.6-6.3	0.245	2.52	1.32-4.78	**0.0049**
>40 and ≤60 years	81	44	71	7	4.4-9.5		1		
>60 years	95	51.6	74	9	6.3-11.6		1.03	0.7-1.53	0.88
**Radionuclide**									
Lutetium-177	29	15.7	21	6	3.8-8.1	0.553	1.50	0.9-2.52	0.12
Yttrium-90	7	3.8	6	7	3.8-6.1		1.37	0.56-3.36	0.50
Combined	148	80.4	124	9	10.2-13.7		1		
Grading									
G1 (Ki67<2%)	29	15.7	21	11	3.6-18.3	0.0662	0.77	0.45-1.32	0.35
G2 (Ki67 3-20%)	75	40.7	57	6	5-6.9		1		
G3 (Ki67>20%)	13	7	13	5	2.3-7.6		0.89	0.45-1.74	0.75
Unknown	67	36.4	60	9	6-11.9		0.60	0.4-0.9	**0.01**
**Previous therapies (other than PRRT)**									
0	5	2.7	2	20	nr	0.856	0.41	0.09-1.81	0.24
1	57	30.9	47	8	6.2-9.7		0.72	0.47-1.12	
2-3	79	42.9	68	6	4.1-7.8		1		
>3	43	23.3	34	8	5.1-10.8		0.69	0.43-1.11	0.12
**Primary tumor**									
Bronchial	15	8.1	13	8	5.1-10.8	0.374	1.31	0.68-2.51	0.43
Pancreas	85	46.1	68	7	4.1-9.9		1		
Small Intestine	36	19.5	27	10	6.8-13.1		0.75	0.44-1.29	0.31
CUP	25	13.6	21	6	0.1-11.8		1.13	0.63-2.02	0.70
Other	23	12.5	22	6	1.4-10.5		1.19	0.71-2.0	0.52
**Functional syndromes**									
Carcinoid syndrome	23	12.5	17	16	7.7-24.2	0.102	0.45	0.25-0.82	0.01
Gastrinoma	10	5.4	8	6	5.3-6.6		1.26	0.58-2.73	0.57
Insulinoma	5	2.7	4	3	0.6-5.9		1.71	0.55-5.36	0.36
Glucagonoma	6	3.2	4	18	4.7-31.2		0.57	0.2-1.69	0.31
VIPoma	4	2.1	3	11	2.9-19		1.48	0.44-4.96	0.54
None	136	73.9	115	7	5.2-8.7		1		

Abbreviations: nr: not reached; 95 CI: 95% confidence interval bold figures indicate significance results.
